# U. S. V. M. A.

**Published:** 1897-04

**Authors:** 


### SOCIETY PROCEEDINGS.

### U. S. V. M. A.

THE resolutions adopted at Buffalo, fittingly recognizing the generous hospitality of the city accorded us in September last, have been suitably engrossed and framed and placed with Chairman Hinkley, of the local committee of U. S. V. M. A. Permission has been asked of the city to allow a photographic copy to be placed in the City Hall.

Every Association meeting throughout the land between now and September should have on its programme, as a feature for consideration, action, aid, and the selection of delegates to the U. S. V. M. A. meeting at Nashville in September.

As the U. S. V. M. A. committee for receiving contributions to the Pasteur Monument Fund is desirous of making a report to the committee at Washington, it is earnestly requested that the members send in their contributions, that our part of this laudable project may be fittingly performed.

President Osgood announces the following appointments for the Committee on Intelligence and Education: Dr. H. D. Gill, Chairman; Drs. Thomas B. Rayner, Olof Schwarzkopf, W. B. Niles, and W. H. Dalrymple.

Dr. Dalrymple expresses himself in the terms of "I am mighty glad Nashville is to be the place of meeting for 1897."

President Osgood should appoint for the Nashville meeting a press committee, a committee on new members, and a local committee of arrangements. There is work for them all, and they could greatly enhance the success of the meeting. We hope he will act on the suggestion.

